# 557 Adverse Events Associated with Fractional CO2 Laser Therapy for Hypertrophic Burn Scars

**DOI:** 10.1093/jbcr/irad045.153

**Published:** 2023-05-15

**Authors:** Paul Won, Michael Cooper, Justin Gillenwater, Haig Yenikomshian

**Affiliations:** University of Southern California, Pasadena, California; University of Southern California Division of Plastic and Reconstructive Surgery, Los Angeles, California; Keck Medicine of USC, Los Angeles, California; University of Southern California, Los Angeles, California; University of Southern California, Pasadena, California; University of Southern California Division of Plastic and Reconstructive Surgery, Los Angeles, California; Keck Medicine of USC, Los Angeles, California; University of Southern California, Los Angeles, California; University of Southern California, Pasadena, California; University of Southern California Division of Plastic and Reconstructive Surgery, Los Angeles, California; Keck Medicine of USC, Los Angeles, California; University of Southern California, Los Angeles, California; University of Southern California, Pasadena, California; University of Southern California Division of Plastic and Reconstructive Surgery, Los Angeles, California; Keck Medicine of USC, Los Angeles, California; University of Southern California, Los Angeles, California

## Abstract

**Introduction:**

Laser therapy is a growing intervention in hypertrophic scar treatment. This therapy is associated with minimal risks, however these are poorly studied in the burn population. Anecdotal reports of adverse effects such as pain and dyspigmentation exist. As patients, stakeholders, and insurance companies decide on reimbursement for this therapy, a better studied safety profile is necessary. This study is a retrospective review of all laser therapies for adverse events.

**Methods:**

A retrospective review of patients who underwent laser therapy at two centers between May 2019 and June 2022. Patient demographics, details of laser treatments, adverse events, and scar characteristics before and after laser therapy were collected.

**Results:**

A total of 167 patients underwent 533 laser treatments. There were 75 (44.9%) males and 92 (55.1%) females, with an average age of 26 (SD: 20.6) years. In total, 94 patients (56.3%) were Hispanic or Latino, 24 (14.4%) Caucasian, and 49 (29.3%) identified as "Other". All patients were treated for hypertrophic scars after burn injuries. For all patients, average number of laser treatments was 3 (SD: 2.2), with a range of 1 to 17 sessions. There were 9 (1.68%) instances of adverse events out of the total laser encounters. There were 5 (0.93%) reports of increased pain, 1 (0.18%) of an open wound, 2 (0.37%) of increased scar discoloration, and 1 (0.18%) of decreased scar pliability. Of the 5 (2.98%) total patients who encountered adverse events, 3 continued with laser therapy. There was no correlation between number of laser treatments and adverse events.

**Conclusions:**

Patients undergoing laser treatment for burn scar management rarely face adverse events, of which the most common were increased scar pain and dyspigmentation. Even so, most patients experiencing adverse events chose to continue treatments. Laser therapy remains effective in burn scar treatment with minimal risks regarding safety profile.

**Applicability of Research to Practice:**

This review will assist patients in making more informed decisions when considering laser therapy for burn scar management.

